# Overexpression the *BnLACS9* could increase the chlorophyll and oil content in *Brassica napus*

**DOI:** 10.1186/s13068-022-02254-3

**Published:** 2023-01-06

**Authors:** Keming Zhu, Nannan Li, Xiangfeng Zheng, Rehman Sarwar, Yulong Li, Jun Cao, Zheng Wang, Xiaoli Tan

**Affiliations:** 1 School of Life Sciences, Jiangsu University, Zhenjiang, China; 2 Chongqing Key Lab of Bioresource for Energy, College of Resources and Environment, Southwest University, Chongqing, China

**Keywords:** *BnLACS9*, Chloroplast, Chlorophyll, Galactolipid, Glycolipid, Oil content

## Abstract

**Background:**

Chlorophyll is a very important pigment involved in photosynthesis, while plant acyl-CoA biosynthesis is derived from plastid-localized fatty acids (FAs). Until now, the regulation of the acyl-CoA pathway for chlorophyll biosynthesis is still unknown.

**Results:**

Here, we identified a long-chain acyl-CoA synthetase (LACS) gene *BnLACS9* from *Brassica napus*. *BnLACS9* complemented a LACS-deficient yeast strain *YB525*, which indicated that BnLACS9 has the LACS function. *BnLACS9* was localized in the chloroplast envelope membrane, while mainly expressed in young leaves and flowers. Overexpression of *BnLACS9* in *Nicotiana benthamiana* resulted in an increase in total CoA and MGDG content. In *B. napus* with overexpression of *BnLACS9*, the number of chloroplast grana lamellae and the chlorophyll content, as well as the MGDG and DGDG contents, increased compared to wild type. The net photosynthetic rate, dry weight of the entire plant and oil content of seeds increased significantly, accompanied by an increase in chlorophyll content. Transcriptome analysis revealed that overexpression of *BnLACS9* improved the pathway of acyl-CoA biosynthesis and further improved the enzymes in the glycolipid synthesis pathway, while acyl-CoA was the substrate for glycolipid synthesis. The increased glycolipids, especially MGDG and DGDG, accelerated the formation of the chloroplast grana lamellae, which increased the number of chloroplast thylakoid grana lamella and further lead to increased chlorophyll content.

**Conclusions:**

In the present study, we demonstrated that BnLACS9 played a crucial role in glycolipids and chlorophyll biosynthesis in *B. napus*. The results also provide a new direction and theoretical basis for the improvement of the agronomic traits of plants.

**Supplementary Information:**

The online version contains supplementary material available at 10.1186/s13068-022-02254-3.

## Introduction

Fatty acids (FAs) are the building blocks of all cellular lipids (including triacylglycerols (TAGs), phospholipids, glycolipids, sterols, and cutins), which are the main energy reservoir of eukaryotic cells and play an essential role in normal plant growth and development. Fatty acid biosynthesis occurs in cytoplasm and plastids. In plastids, free FAs covert to acyl-CoAs (16: 0 CoA, 18: 0 CoA and 18: 1 CoA) by the long-chain acyl-CoA synthetase (LACS, EC 6.2.1.3), and then exported to the endoplasmic reticulum (ER) through the cytosol for generating TAGs [1–5].

There are multiple LACS genes in plants, which encode enzymes that perform different roles in lipid metabolism [4, 6–8]. In *Arabidopsis*, nine genes (*AtLACS1*, *AtLACS2*, *AtLACS3*, *AtLACS4*, *AtLACS5*, *AtLACS6*, *AtLACS7*, *AtLACS8*, *AtLACS9*) that encode LACS have been identified [4, 9]. Amino acid sequence analysis found they all have one very highly conserved motif, the AMP-binding protein (AMPBP) superfamily [10]. Seven of the nine genes could complement the growth phenotype of a LACS-deficient yeast strain, YB525, except *AtLACS6*, and *AtLACS7* [4, 11]. *AtLACS1* and *AtLACS2* were located in ER and the *AtLACS1* has overlapping functions with *AtLACS2* in wax and cutin synthesis in *Arabidopsis thaliana* [12–14]. Dirk Jessen found that *AtLACS1* and *AtLACS4* play a synergistic effect in the proper formation of the pollen coat in *Arabidopsis* [15]. Furthermore, *AtLACS4* could catalyze the first step in conversion of peroxisomal indole-3-butyric acid to IAA [16]. *AtLACS6* and *AtLACS7* play important roles in activating FAs for β-oxidation in the peroxisome [17, 18]. It is known that *AtLACS9* exists in the chloroplast, a major contributor to chloroplastic LACS activity, involved in the export of plastidial FA export for TAG formation, and its function partially overlaps with *LACS1* and *LACS4* in *Arabidopsis* seed oil biosynthesis [19–21]. However, the function of *AtLACS9* is still controversial, a study showed that *AtLACS9* might help transport lipids from the ER back to the plastid [22].

*Brassica napus* (*B. napus*) is a worldwide oil crop, which is one of the important edible oils for human consumption and as a raw material for the biofuel and pharmaceutical industry [23–25]. Therefore, increasing the oil content of seeds is very important for geneticists and breeders, and has become a major subject of oil crop research [26–28]. In *B. napus*, LACSs play pivotal roles in lipid biosynthesis and oil accumulation [7, 29–31]. Pongdontri et al*.* reported that *ACS6* was strongly expressed in embryos of rapeseed and could improve the efficiency of lipid synthesis [30]. The heterologous expression of the *BnLACS4* gene in yeast cells could increase the content of C16: 0 and C18: 0 by 45.7 and 21.7%, respectively [31]. Overexpression of *BnLACS2* in yeast and rapeseed could increase oil content, and BnLACS2 was located in the ER [29]. Xiao et al. identified 34 *BnLACSs* by a comprehensive genome-wide analysis of the gene family in *B. napus*. Comparative expression analysis between high- and low-oil *B. napus* cultivars revealed that BnaLACS6-4, BnaLACS9-3, and BnaLACS9-4 may be involved in chloroplast fatty acid synthesis, and BnaLACS1-10 and 4-1 may play a vital role in lipid biosynthesis [7]. However, except *BnLACS2* and *ACS6*, the functions of other *LACS* genes are still not very clear in *B. napus*.

In this study, the *BnLACS9* was isolated from developing rape embryos, its cDNA encoding a novel acyl-CoA synthase. Our results showed that overexpression of *BnLACS9* in *Nicotiana benthamiana* can increase the content of galactolipids in the leaf accompanied by an increase in chlorophyll content. Overexpression of *BnLACS9* in *B. napus* also caused the chlorophyll content to be upgraded significantly compared with the wild type. So, our data provide a new insight into the pathway of chlorophyll biosynthesis. The *BnLACS9* can regulate the content of the chlorophyll by influencing the chloroplast biosynthesis through regulating the chloroplast functional lipid biogenesis. The photosynthetic rate is directly related to the chlorophyll content of the plant, so we take a new way to increase the biomass of *B. napus* by enhancing the expression of the *BnLACS9* gene in some way.

## Results

### BnLACS9 is highly homologous with AtLACS9

**Fig. 1:**
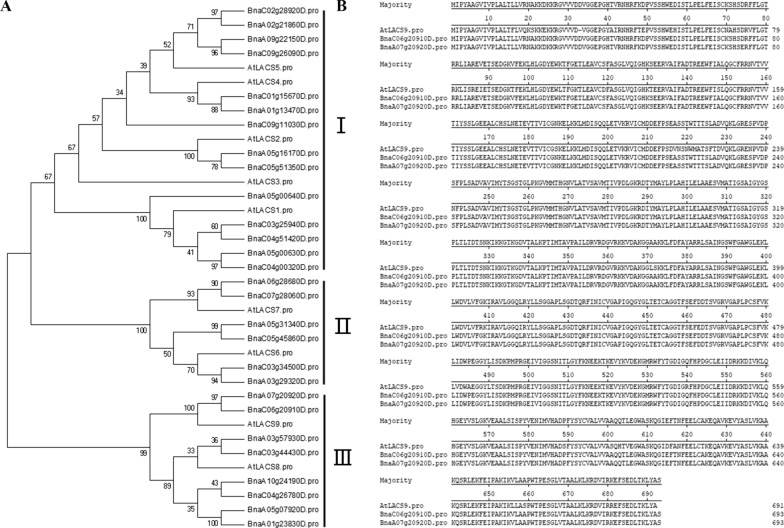
Analysis of the amino acid sequences of BnLACS and AtLACS. **A** The phylogenetic tree of the LACS members was constructed MEGA 4.0. The Arabic numerals represent the credibility; Latin numbers: LACS classification according to the evolutionary distance; **B** multiple sequence alignments of the LACS9 amino acid sequences from *B. napus* and *A. thaliana*

In *Arabidopsis thaliana*, nine long-chain acyl-CoA synthetases (LACSs) belong to a large superfamily of acyl-activating enzymes [4, 9], and are involved in FA transport. To search the homologous proteins in *B. napus* L., the amino acid sequences of AtLACS1-9 (AT2G47240, AT1G49430, AT1G64400, AT4G23850, AT4G11030, AT3G05970, AT5G27600, AT2G04350, AT1G77590) were used as the query probe to search in *Brassica* genome sequences databases (http://www.genoscope.cns.fr/blat-server/cgi-bin/colza/webBlat). In total, 29 significant homologous proteins were obtained (Additional file 5: Table S1). The phylogenetic tree analysis was performed based on the similarities of the conserved domain sequences among these proteins. As shown in Fig. 1A, except AtLACS3, other AtLACSs have 2 or 4 homologous proteins in *B napus*, because the *B. napus* is allopolyploidy, and it has a duplicate genome [32]. All LACS proteins could be divided into three branches, LACS1 to LACS5 formed the first branch, LACS6 and LACS7 formed the second branch, and LACS8 and LACS9 formed the third branch (Fig. 1A), showing that these LACS proteins could have a different function. The phylogenetic tree analysis showed that AtLACS9, BnaA07g20920D (BnLACS9-A07), and BnaC06g20910D (BnLACS9-C06) were in one clade (Fig. 1A), and they share 91.6% and 91.9% similarity in amino acid sequence, respectively (Fig. 1B). AtLACS9, BnLACS9-A07 and BnLACS9-C06 are closely related in terms of amino acid sequence and evolutionary relationship, which implies that they likely have similar functions. The similarity between BnLACS9-A07 and BnLACS9-C06 was as high as 99.7%, so we selected BnLACS9-C06 as the target gene in this study.

To determine whether BnLACS9 has LACS, a yeast vector pYES2-BnLACS9 was constructed and analyzed by a yeast complementary expression system. Yeast strain *YB525* is a strain with affected LACS activity [33], and this defective yeast cannot grow in the medium only using FA as the sole carbon source. A yeast complementarity test was used to determine the growth of yeast cells transferred into different vectors in a drop-out medium with different FAs as the sole carbon source for 72 h. *YB525* yeast cells transferred to the empty vector pYES2 could not grow in a drop-out medium with different FAs as the sole carbon source. While the *YB525* yeast cells transferred into the pYES2-BnLACS9 vector grow better and has different growth rates in drop-out medium with different FAs as the sole carbon source (Additional file 1: Fig. S1). The OD (optical density) value of *YB525* yeast cells transferred into the pYES2-BnLACS9 vector could reach to 0.38 in a drop-out medium with only fatty acid C18: 0 as the sole carbon source, while the OD value of yeast 0.05 in drop-out medium with only fatty acid C12: 0 as the sole carbon source (Additional file 1: Fig. S1). These results indicate that the heterologous expression of the *BnLACS9* gene can complement the deficient yeast and has the activity of LACS protease.

### Transient expression of BnLACS9 in N. benthamiana leaves could enhance the content of FA, MGDG and total chlorophyll

In order to further study the function of the *BnLACS9* gene, *BnLACS9* was transiently expressed in *N. benthamiana* leaves. In plants, FAs are uniquely synthesized in plastids [34]. FA compositions from *N. benthamiana* leaves of both accessions were determined by gas chromatography–mass spectrometry (GC–MS). From Additional file 6: Table S2, we could find that the transient expression of the *BnLACS9* gene in leaves of *N. benthamiana* can increase the content of FA, especially the C16: 0 and C18: 2, their contents were 221.3034 ± 14.0447 avfmol/mg and 59.2165 ± 9.703042 avfmol/mg, respectively. While the C16:0 and C18:2 are 134.9407 ± 7.264136 avfmol/mg and 23.56197 ± 3.008565 avfmol/mg in the control, respectively (Additional file 6: Table S2). As a result, the total FA content in the transient expression of the *BnLACS9* gene in *N. benthamiana* leaves was 574.5768 ± 35.79813 avfmol/mg, while that of control was only 395.4662 ± 13.63986 avfmol/mg. These results indicated that *BnLACS9* could increase the FA content in *N. benthamiana* leaves.

**Fig. 2:**
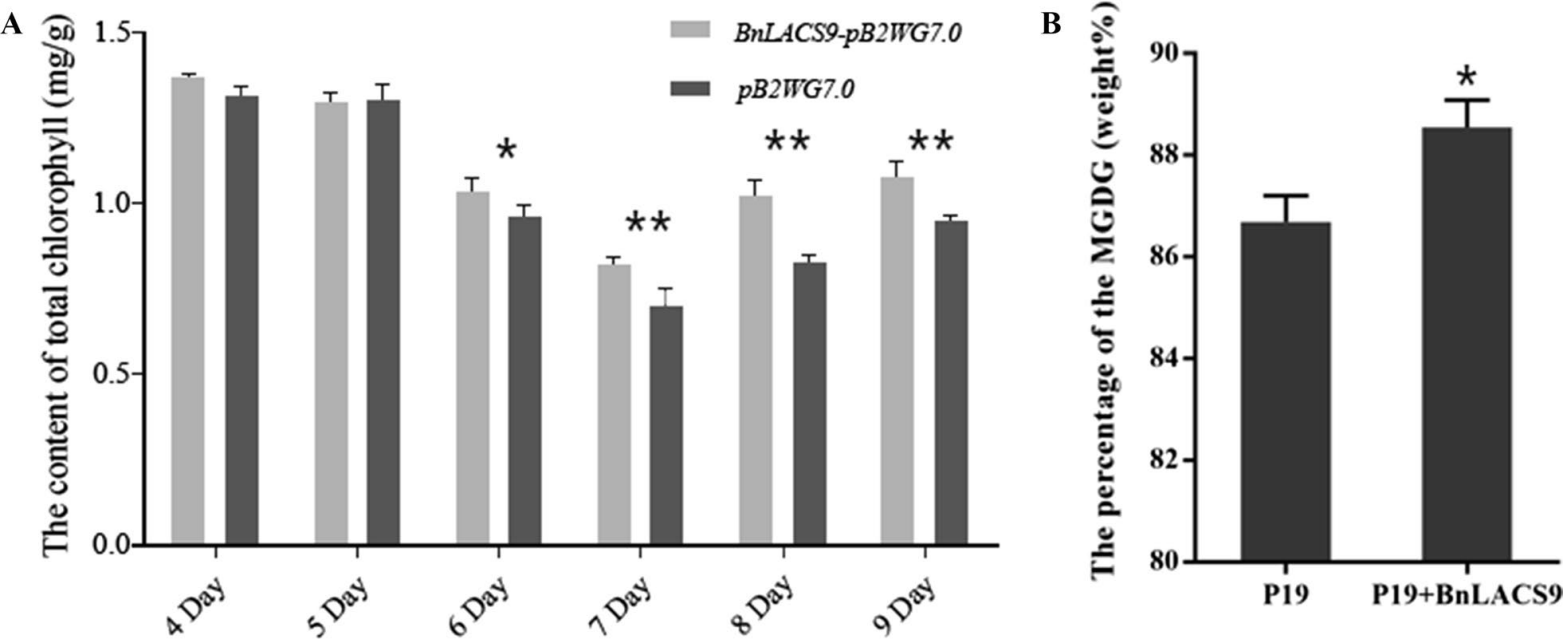
The content of chlorophyll and MGDG in the leaves of *N. benthamiana.* **A** The content of the chlorophyll. Gray: the leaves overexpression of *BnLACS9*; black: the control. **B** The content of the MGDG. *p < 0.05, **p < 0.01, ***p < 0.001. Student’s *t*-test was used to generate *p*-value

We also detected the chlorophyll content in *N. benthamiana* leaves of transient expression of *BnLACS9* and its control. We found that within 5 days after the *BnLACS9* gene was transferred into *N. benthamiana* leaves, there was no significant difference in chlorophyll content between the two groups. From the sixth day, the chlorophyll content of *N. benthamiana* leaves transfected with the *BnLACS9* gene was significantly higher than that of wild type, and reached the maximum on the eighth day (Fig. 2A). Monogalactosyl-diaclyglycerol (MGDG) is one of the main components of the chloroplast photosynthetic membrane [34]. We also detected the MGDG content in the leaves of *N. benthamiana* between the transient expression of *BnLACS9* and its control. The content of MGDG in *N. benthamiana* leaves transfected with the *BnLACS9* gene was significantly higher than that of the control (Fig. 2B). All these results indicated that BnLACS9 could improve the content of FA, MGDG and total chlorophyll.

### The expression pattern and subcellular localization of BnLACS9

To better understand the function of BnLACS9, we examine the expression pattern of *BnLACS9* in various organs, including the root, stem, young leaf, old leaf, flower, and silique of Ningyou 12 (NY12). We performed quantitative real-time PCR (qPCR) analysis to estimate the level of the *BnLACS9* transcript. *BnLACS9* is mainly expressed in young leaves and flowers (Additional file 2: Fig. S2). We could detect the expression of the *BnLACS9* gene in other organs, but the lowest expression in the old leaf. These results show that the *BnLACS9* gene is temporally and spatially expressed.

**Fig. 3:**
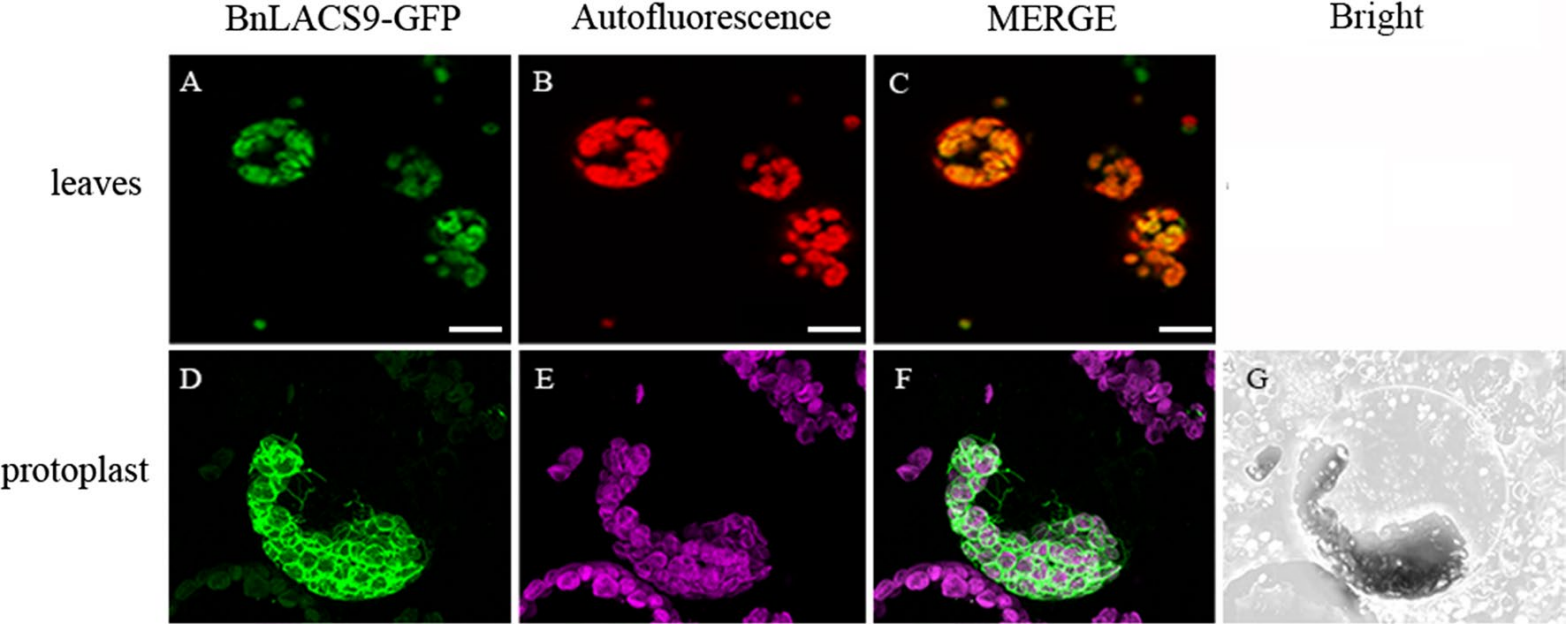
The subcellular localization of BnLACS9 protein. Confocal microscopic analysis after 35S-promoter driven in planta expression of GFP-fusion proteins. GFP fluorescence (GFP) is shown in green, chlorophyll autofluorescence (chlorophyll) in red/purple, and an overlay (merge) of representative protoplasts are shown. **A**–**C** Expression pattern in *N. benthamiana* leaves; **D**–**G** expression pattern in *N. benthamiana* protoplasts. Size bars: 10 μm

Localization studies were further carried out on BnLACS9 to understand its mechanism of action. To determine the subcellular location of BnLACS9, GFP (green fluorescent protein) was fused to the C terminus of BnLACS9 under the control of a 35S promoter and the fusion gene was transformed into leaves of *N. benthamiana*. We found that the expression pattern of BnLACS9-GFP overlapped with the chlorophyll autofluorescence in *N. benthamiana* leaves (Fig. 3A–C), suggesting that BnLACS9 may be targeted at the chloroplasts. To further study the location of BnLACS9-GFP in chloroplasts, we expressed 35S:BnLACS9-GFP in *N. benthamiana* leaves for subsequent protoplast isolation and observed the protoplasts of *N. benthamiana*. From Fig. 3E–G, we observed circular structures of the fusion protein fluorescence signal around the chloroplast, but did not overlap with the spontaneous fluorescence signal of the chloroplast, and even formed loop structures and thin tubules from the chloroplast. With all these observations, we could conclude that BnLACS9 might be located on the chloroplast envelope membrane.

### Overexpression of BnLACS9 in B. napus increased the content of the oil content

Previous studies showed that the *BnLACS9* gene has an activity of LACS protease (Additional file 1: Fig. S1) and could increase the FA content in *N. benthamiana* leaves (Additional file 6: Table S2). To study the *BnLACS9* gene in rapeseed plant, transgenic rapeseed plants overexpressing *BnLACS9* were generated (Additional file 3: Fig. S3). Homozygous *BnLACS9-*overexpressed lines (#6, #12 and #18) were selected from the positive transgenic plants to evaluate the function of *BnLACS9*.

**Fig. 4:**
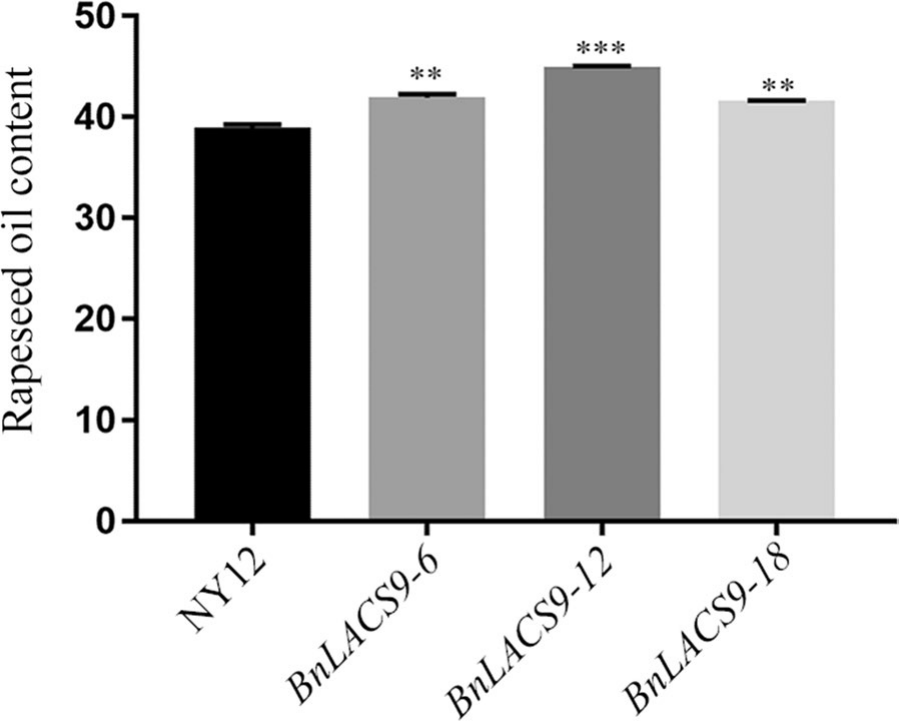
Rapeseed oil content was determined by NMR. *p < 0.05, **p < 0.01, ***p < 0.001. Student’s *t*-test was used to generate the *p*-value

The oil content of *BnLACS9*-overexpressed plant seeds was determined by nuclear magnetic resonance (NMR). As shown in Fig. 4, the oil content of NY12 was only 39.7%, while the oil content of the three overexpression lines exceeded 40%, of which the oil content of *BnLACS9*-12 reached 45.64%. In addition, the seed oil content of overexpressed *BnLACS9* plants was determined by near-infrared (NIR) (Additional file 7: Table S3), and the results also showed the same with NMR. The results of the two methods were consistent, and the oil content of rape seeds overexpressing *BnLACS9* increased.

### Overexpression of BnLACS9 in B. napus could enhance the content of total chlorophyll

**Fig. 5:**
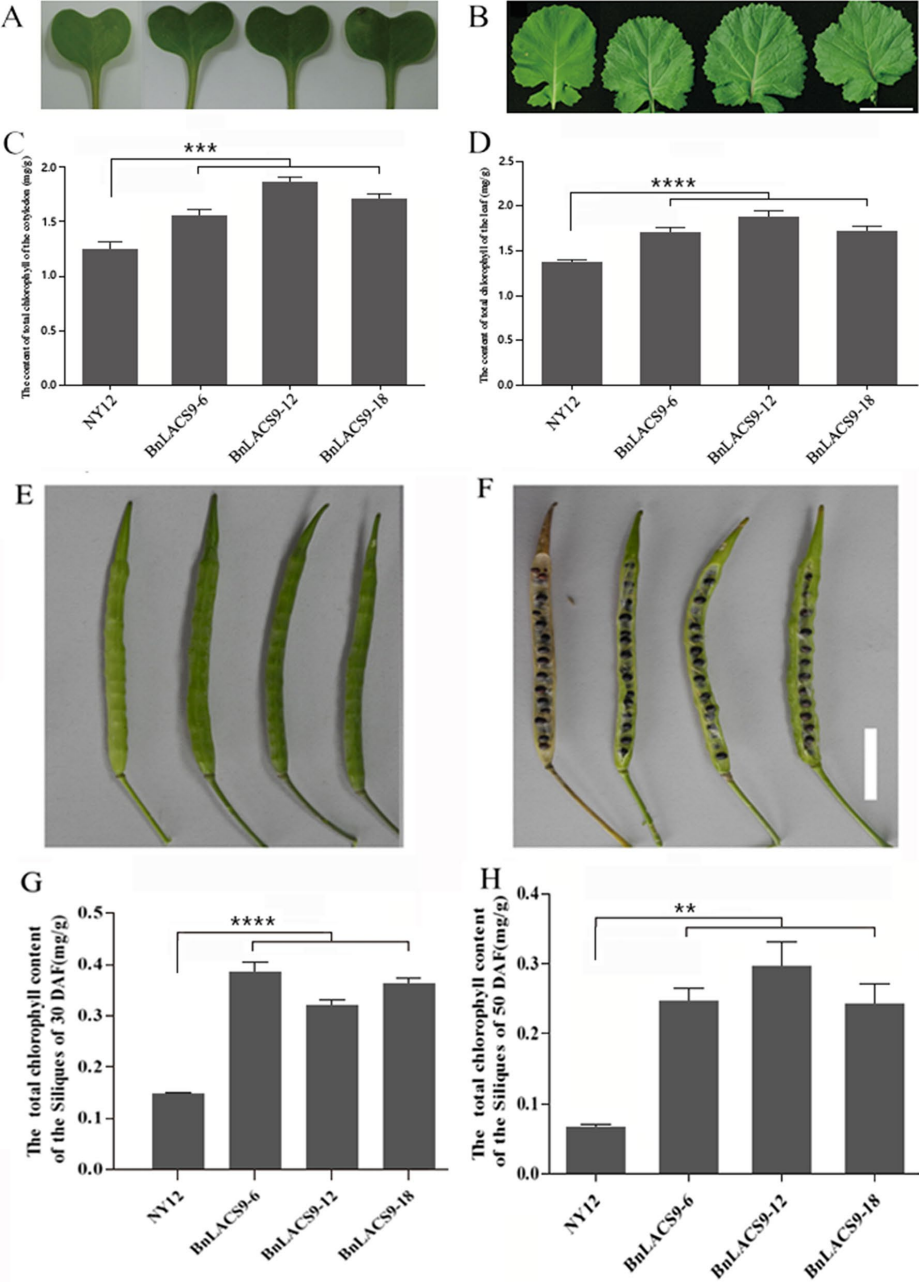
Analysis of the chlorophyll content of the leaf and the siliques. **A** The phenotype of cotyledons in 10 days after germination; **B** the phenotype of leaves in thirty days after germination; **C** the chlorophyll content of cotyledons in 10 days after germination; **D** the chlorophyll content of leaves in 30 days after germination. Size bars: 1 cm. **E** The phenotype of siliques in 30 DAF (days after flowering); **F** the phenotype of siliques in 50 DAF; **G** the chlorophyll content of the siliques in 30 DAF; **H** the chlorophyll content of the siliques in 50 DAF. Size bars: 1 cm. *p < 0.05, **p < 0.01, ***p < 0.001. Student’s *t*-test was used to generate the *p*-value

The oil content of *BnLACS9*-overexpressed plant seeds was increased, we also found that the cotyledons and leaves of *BnLACS9-*overexpressed plants were significantly greener than those of the wild type (Fig. 5A, B). The chlorophyll content of *BnLACS9-*overexpressed plants was also higher than that of wild type in 7-day-old cotyledons and 30-day-old leaves (Fig. 5C, D). We also found that the siliques of *BnLACS9-*overexpressed plants could delay senescence compared with the control (Fig. 5E, F). So, we measured the chlorophyll content of the silique at 30 DAF (days after flowering) and 50 DAF and showed that the chlorophyll content in overexpressed plants was significantly higher than that in wild-type plants (Fig. 5G, H). These results indicated that overexpression of *BnLACS9* gene can increase chlorophyll content and delay silique senescence in *B. napus*.

**Fig. 6:**
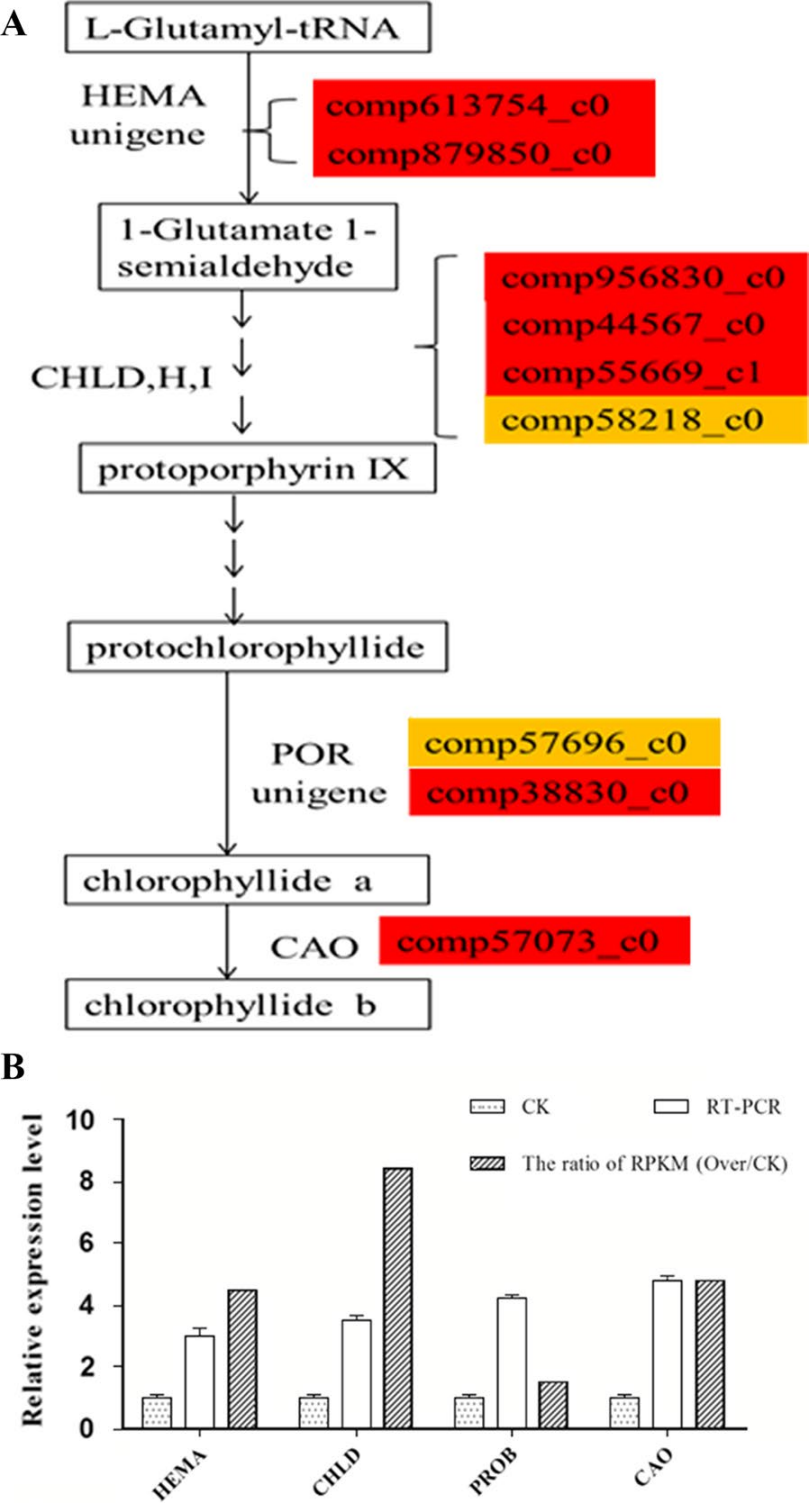
The relative expression level of key genes in chlorophyll biogenesis by transcriptome sequencing (**A**) and by RT-PCR (**B**). **A** ‘comp’ represents the unigene number. The box colors represent the relative expression level of the unigene in the *BnLACS9* overexpression plants. Red box: the unigene relative expression level is two times more than the wild type; yellow box: the relative expression levels are between 1.5 and 2 times more than the wild type. HEMA, glutamyl tRNA reductase; CHLD,H,I, Mg-chelatase (D,H,I) subunit; POR, NADPH-protochlorophyllide oxidoreductase; CAO, Chlorophyll a oxygenase. **B** CK: wild type; RT-PCR: the result of RT-PCR identification; the ratio of RPKM (Over/CK): the relative expression level of transcriptome sequencing. *p < 0.05, **p < 0.01, ***p < 0.001. Student’s *t*-test was used to generate *p*-value

In order to characterize the mechanism that *BnLACS9* influenced the content of chlorophyll through regulating the genes in the pathway of the chlorophyll synthesis, we sequenced the transcriptome of the leaves of the overexpression of *BnLACS9* plants and wild type. The unigene was shown as ‘comp…_c0’. The expression level of the unigenes was represented by RPKM (Reads Per Kilobase per Million mapped reads) [37]. Transcriptome analysis showed 9 genes (comp613754_c0, comp879850_c0, comp44567_c0, comp956830_c0, comp55669_c1, comp58218_c0, comp57696_c0, comp38830_c0, comp57073_c0) in chloroplast synthesis pathway were up-regulated in *BnLACS9-*overexpressed plants (Fig. 6). We confirmed the transcript levels of genes *HEMA* (Glutamyl tRNA reductase), *CHLD* (Mg-chelatase D subunit), *PORB* (NADPH-protochlorophyllide oxidoreductase B), *CAO* (Chlorophyll a oxygenase) by RT-PCR (reverse transcription-polymerase chain reaction, RT-PCR) (Fig. 6B), and showed that these genes were all up-regulated. These results suggested the overexpression of *BnLACS9* upgraded the key genes of the chlorophyll synthesis pathway and further increased the content of the chlorophyll.

### Overexpression of BnLACS9 in B. napus could increase the number of thylakoid layer structures in chloroplast and the content of the galactolipids

**Fig. 7:**
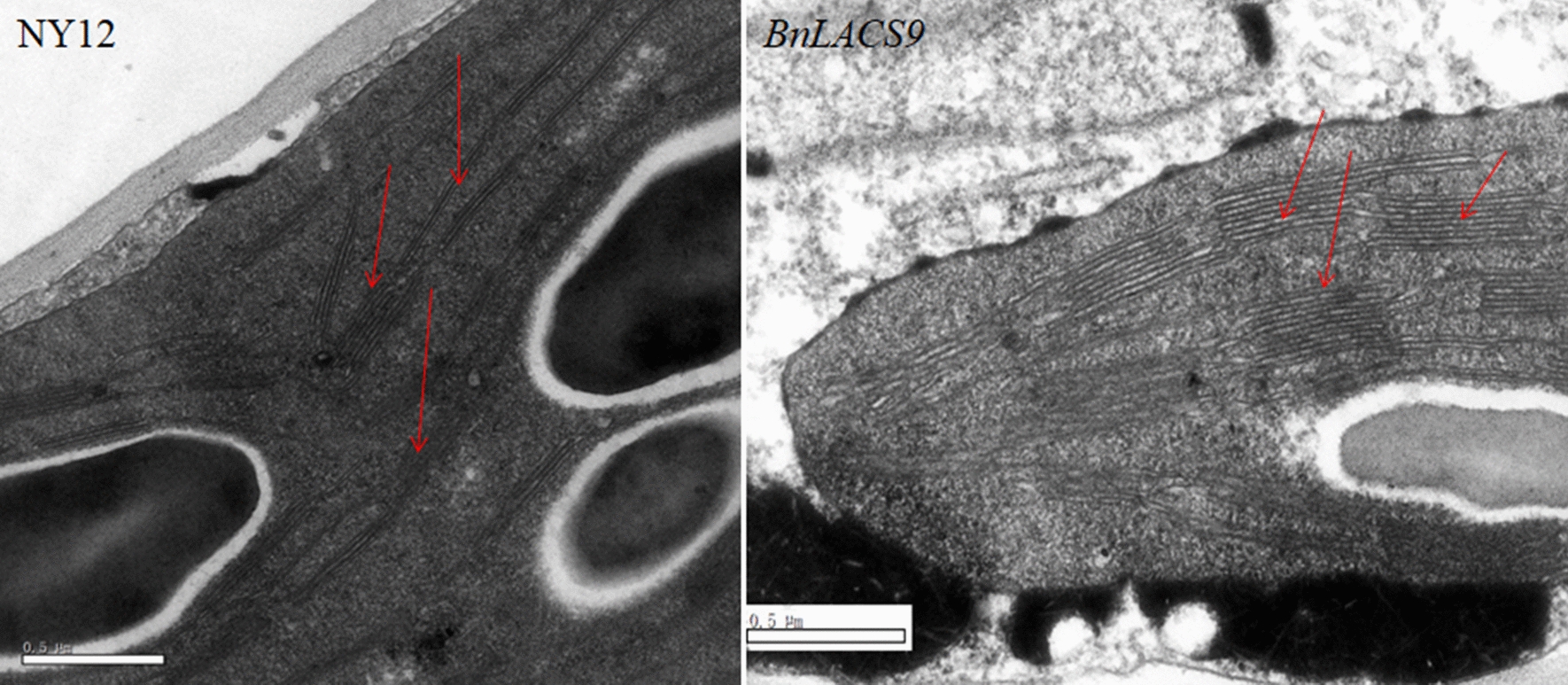
Ultrastructural differences of chloroplast thylakoid between wild-type (**A**) and overexpression plants of *BnLACS9* (**B**). **A** The ultrastructure of chloroplast thylakoid of wild type; **B** the ultrastructure of chloroplast thylakoid of the *BnLACS9* overexpression plants. Red arrows indicate the layers of the thylakoid grana. Size bars: 0.5 μm

The *BnLACS9-*overexpressed plants had higher chlorophyll content (Fig. 5), so we observed the chloroplast structure by transmission electron microscopy (TEM). Ultrastructural observation showed that the number of thylakoid grana slice layers in *BnLACS9-*overexpressed plants was more than that in wild-type plants (Fig. 7). There were fewer layers of thylakoid grana (2–3 layers) were observed in NY12, while more layers of thylakoid grana (5–8 layers) were observed in plants overexpressed with *BnLACS9* (Fig. 7). These results indicated that overexpression of *BnLACS9* gene could increase the number of thylakoid grana slice layer, make leaves greener and have higher chlorophyll content.

Previous studies showed that the main lipid component of the thylakoid layer structure such as monogalactosyldiacylglycerol (MGDG), digalactosyldiacylglycerol (DGDG), phosphatidylglycerol (PG) and sulfoquinovosyldiacylglycerol (SQDG) [2, 35, 36]. Therefore, we used thin-layer chromatography to analyze the lipid content in leaves. The contents of MGDG, DGDG, PG, and SQDG in *BnLACS9-*overexpressed lines were higher than those in wild type, only PG was not detected in *BnLACS9-*overexpressed line 6 (Additional file 4: Fig. S4).

**Fig. 8:**
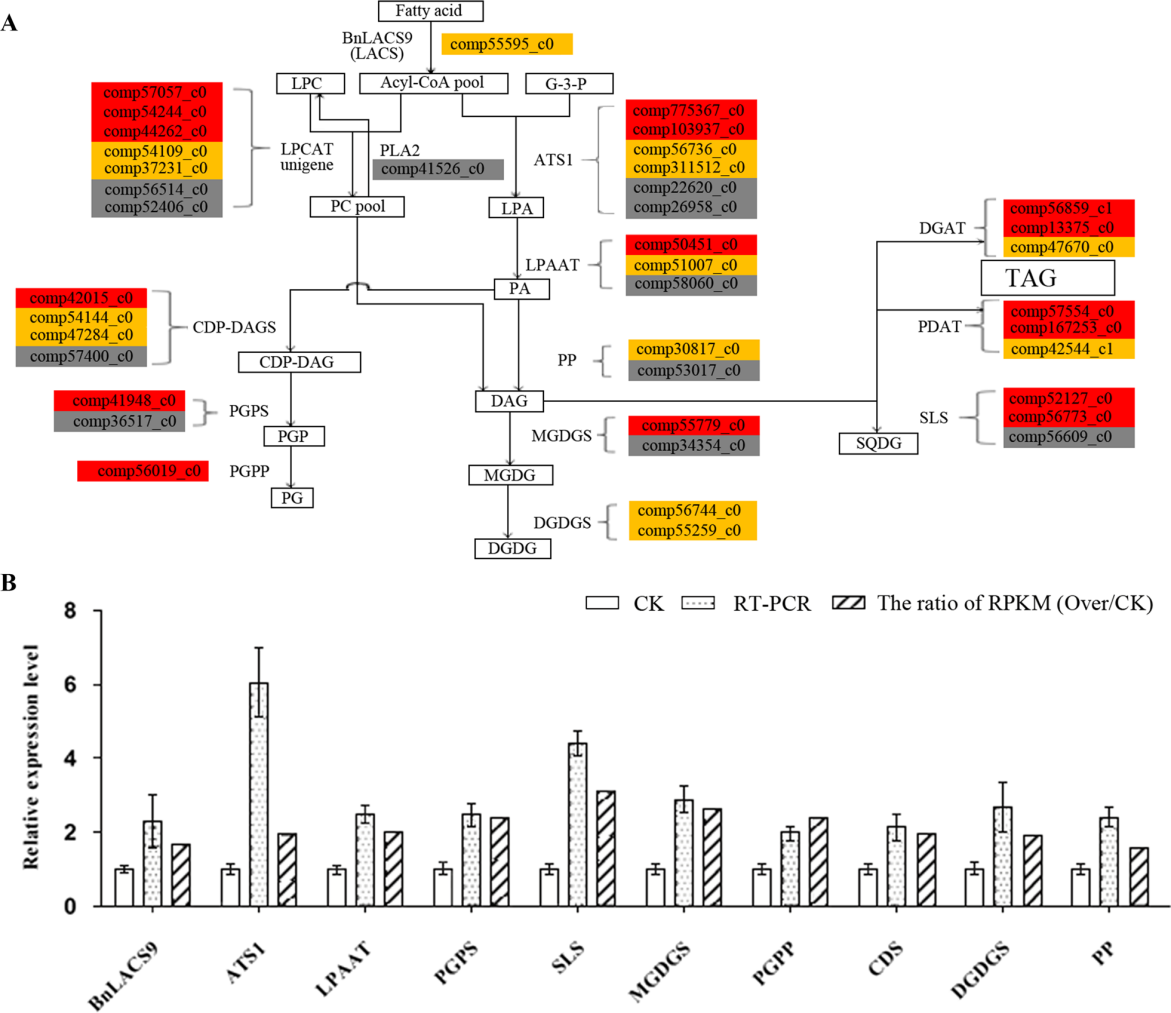
The relative expression level of the genes in lipid synthesis by transcriptome sequencing (**A**) and by RT-PCR (**B**). **A** ‘comp’ represents the unigene number. The box colors represent the relative expression level of the unigene in the *BnLACS9* overexpression plants. Red box: the unigene relative expression level is two times more than the wild type; yellow box: the relative expression levels are between 1.5 and 2 times; gray box: the relative expression levels change a bit. **B** CK: wild type; RT-PCR: the result of RT-PCR identification; the ratio of RPKM (OVER/CK): the relative expression level of *BnLACS9*, *ATS1*, *LPPAT*, *PGPS*, *SLS*, *MGDGS*, *PGPP*, *CDS*, *DGDGS* and *PP* from transcriptome sequencing data

In order to make a thorough inquiry about how the content of the glycolipid was increased in the overexpression of *BnLACS9* plants and to elucidate the gene regulatory network of the pathway. Transcriptomes in the overexpression of the *BnLACS9* line and wild type were analyzed. The unigenes related to glycolipid synthesis were picked from the transcriptome data of *B. napus*. The unigene was shown as ‘comp…_c0’. Transcriptome studies showed that most unigenes of glycolipid biosynthesis were up-regulated in the *BnLACS9-*overexpressed plants (Fig. 8A). To confirm the result, nine unigenes were chosen to verify by RT-PCR. These unigenes were *ATS1* (glycerol-3-P acyl-ACP acyltransferase) (comp_775367c0), *LPAAT* (Lysophosphatidic acid acyltransferase) (comp50451_c0), *PP* (phosphatidate phosphatase) (comp30817_c0), *PGPS* (phosphatidylglycerol phosphate synthase) (comp41948_c0), *PGPP* (phosphatidylglycerol phosphate phosphatase) (comp56019_c0), *MGDGS* (monogalactosyldiacylglycerol transferase) (comp55779_c0), *DGDGS* (UDP-galactose-dependent digalactosyldiacylglycerol synthase) (comp56744_c0), *SLS* (sulfolipid synthase) (comp52127_c0), *CDP-DAGS* (cytidine diphosphate-diacylglycerol synthase) (comp42015_c0). RT-PCR results showed that *ATS1*, *LPAAT*, *PP*, *PGPS*, *PGPP*, *PP*, *MGDGS*, *DGDGS*, *SLS* and *CDP-DAGS* had higher expression in the overexpression of *BnLACS9* plant than the wild type (Fig. 8B). In particular, the expression level of the *ATS1* and *SLS* is four times higher more than the CK; and other genes in overexpression of *BnLACS9* plant is exceeded double than the CK (Fig. 8B), these results were similar with transcriptomes data. Taken together, the results suggest that *BnLACS9* regulated the expression of genes in the pathway of glycolipid biosynthesis. The higher expression level of these genes increased the content of the glycolipid.

### Overexpression of BnLACS9 in B. napus could increase photosynthetic efficiency and dry weight of rapeseed

**Fig. 9:**
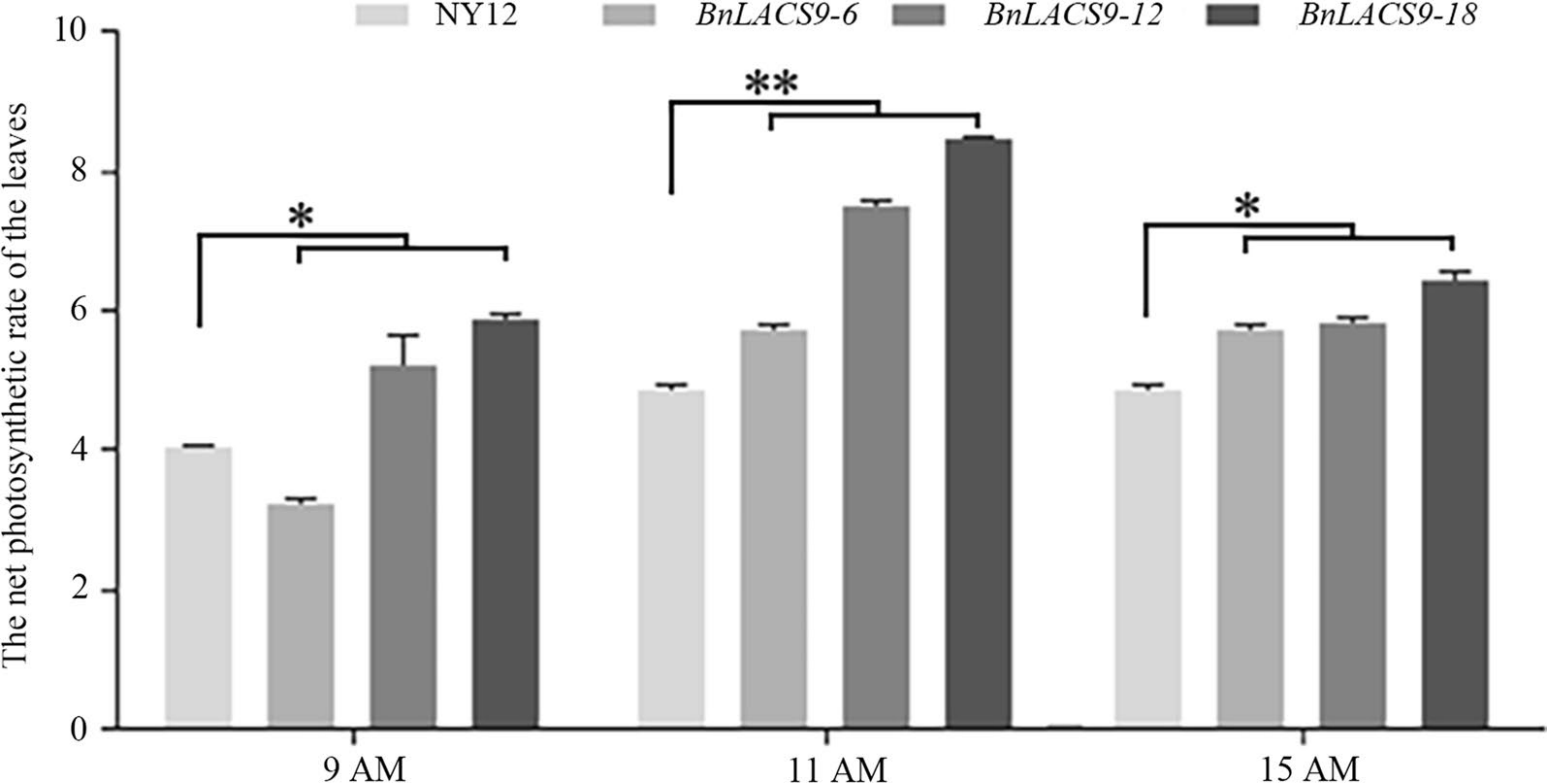
The net photosynthetic rate of leaves of *BnLACS9* overexpression transgenic lines. *p < 0.05, **p < 0.01. Student’s *t*-test was used to generate the *p*-value

**Fig. 10:**
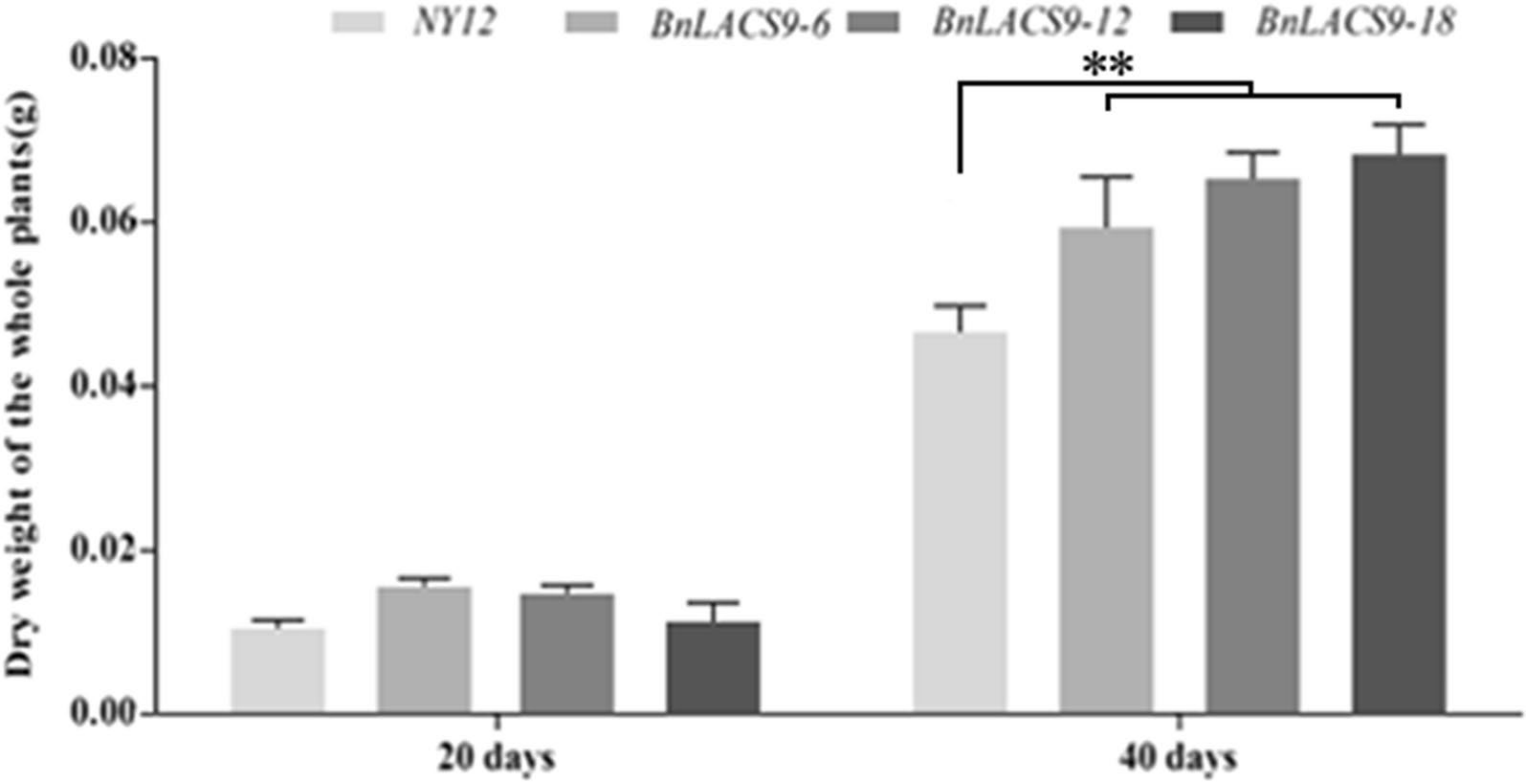
The dry weight of the seedling from *BnLACS9* overexpression transgenic lines. *p < 0.05, **p < 0.01. Student’s *t*-test was used to generate the *p*-value

Previous studies have shown that overexpression of *BnLACS9* could improve the chlorophyll content in rapeseed (Fig. 5), and increase the expression of genes in the chlorophyll synthesis pathway (Fig. 6). So, we measured the photosynthetic efficiency of leaves of overexpressed plants and wild-type plants. We found that the photosynthetic efficiency of the leaves of the overexpressed BnLACS9 plants was significantly higher than that of wild-type plants (Fig. 9). We measured the dry weight of the *BnLACS9*-overexpressed lines at 20 and 40 days after seed germination. At 20 days, we could see that the dry weights have been increased. On the 40th day after germination, the dry weight of *BnLACS9*-overexpressed lines increased significantly (Fig. 10). From the above, overexpression of *BnLACS9* in plants could increase photosynthetic efficiency and the biomass of the plant in rapeseed.

## Discussion

For a long time, increasing the oil content of oilseed crops has been the core problem of oilseed breeding [25, 34, 37, 38]. LACSs have been show to play a key role in FA and lipid metabolism and could increase oil content [4, 11, 12, 29]. However, there have been few studies on the function of LACS proteins in *B. napus*, a major oil crop in the world. In this study, we characterized the function of *BnLACS9* in *B. napus*. We showed that BnLACS9 has LACS activity, which could complement a LACS-deficient yeast strain (*YB525*). Moreover, overexpression of *BnLACS9* in *B. napus* could increase the content of chlorophyll by regulating the pathway of glycolipid synthesis, and then increases lipid content. Therefore, *BnLACS9* is a candidate gene for high-oil-content breeding in rapeseed.

The *B. napus* (an allotetraploid) was formed by spontaneous hybridization between *B.* rape and *B. oleracea* about 7500 years ago [39], which has a highly homologous genome with *Arabidopsis thaliana* [40]. Xiao et al. found that there are 4 *AtLACS9* homologous genes (*BnaLACS9-1*, *9-2*, *9-3*, and *9-4*) in rapeseed, however *BnaLACS9-1* and *9-2* lost gene function because they obviously different from other *BnaLACSs* and hardly expressed in all tissues [7]. Our phylogenetic analyses showed that BnaA07g20920D (BnLACS9-A07) and BnaC06g20910D were homologous to *Arabidopsis* AtLACS9, and share 91.6% and 91.9% similarity in amino acid sequence, respectively (Fig. 1), speculated that they have similar functions. Previous studies have shown that most of the *LACS* genes have the ability to complement a strain of yeast lacking LACS [4]. Our study showed that heterogeneous expression of *BnLACS9* can complement the LACS-deficient yeast mutant *YB525*, which could rescue with long-chain FAs (C14-C22) as the sole carbon source, but not with short chain FA (C12) (Additional file 1: Fig. S1). These results showed that *BnLACS9* has the long-chain acyl-CoA synthetase activity, and also revealed its substrate preference and specificity.

It is well known that the function of protein is closely related to its location. In *Arabidopsis*, previous studies have shown that LACS1, 4 and 8 are located in the ER, LACS6 and 7 are located in the peroxisome, and LACS9 resides in the plastid envelope [4, 12, 19]. Breuers et al. further research demonstrated the localization of AtLACS9 to the outer envelope membrane [41]. In rice, the OsLACS9 was located in the chloroplast envelope membrane [42]. In this study, subcellular localization analysis indicated that the fluorescence signal of the BnLACS9-GFP fusion protein was around the chloroplast but did not overlap with the spontaneous fluorescence signal of the chloroplast and even formed loop structures and thin tubules from the chloroplast (Fig. 3). The discovery of the loop structure suggests that BnLACS9 has a function at the contact site between organelles, such as the chloroplast and the ER. Therefore, it helps to transport acyl-CoA formed in chloroplasts to ER, and then form TAGs. This is consistent with the fact that ACSL4 is present in the mitochondrial-associated membrane in animal cells [43].

In plant, activation of free FAs to acyl-CoA derivatives is necessary to provide the substrates for glycolipid biogenesis. The synthesis of glycolipid can be divided into chloroplast and endoplasmic reticulum: the inner envelope-localized prokaryotic pathway and the ER-localized eukaryotic pathway [44]. Chloroplast LACS activity is essential to for glycolipid synthesis, since chloroplasts are the main site of de novo FA synthesis [45]. The AtLACS9*,* which has been shown to reside in the plastid [46], is considered to be the main LACS isoform involved in the production of acyl-CoA for the biosynthesis of membrane glycerolipids and storage TAGs [47]. The BnLACS9 is belonged to acyl-CoA synthase, transform the FA into acyl-CoA (Fig. 4). Overexpression of *BnLACS9* stimulated much more FAs from chloroplast, which are further used for the synthesis of glycerolipids, so that the contents of MGDG, DGDG, PG, and SQDG in *BnLACS9-*overexpressed lines were higher than those in wild type (Additional file 4: Fig. S4), and *BnLACS9-*overexpressed plants had more thylakoid grana slice layers than wild type (Fig. 7). These results were also confirmed by transcriptome and RT-PCR studies: most glycolipid biosynthesis genes were up-regulated in plants overexpressed with BnLACS9 (Fig. 8). Furthermore, plants overexpressed with BnLACS9 were significantly greener, their chlorophyll content increased (Fig. 5), and chlorophyll synthesis genes were up-regulated (Fig. 6). Therefore, BnLACS9-overexpressed plants have a higher photosynthetic efficiency (Fig. 9), which is conducive to the accumulation of dry matter (Fig. 10) and the formation of oil (Fig. 2). In conclusion, BnLACS9 is a key gene in oil synthesis.

**Fig. 11:**
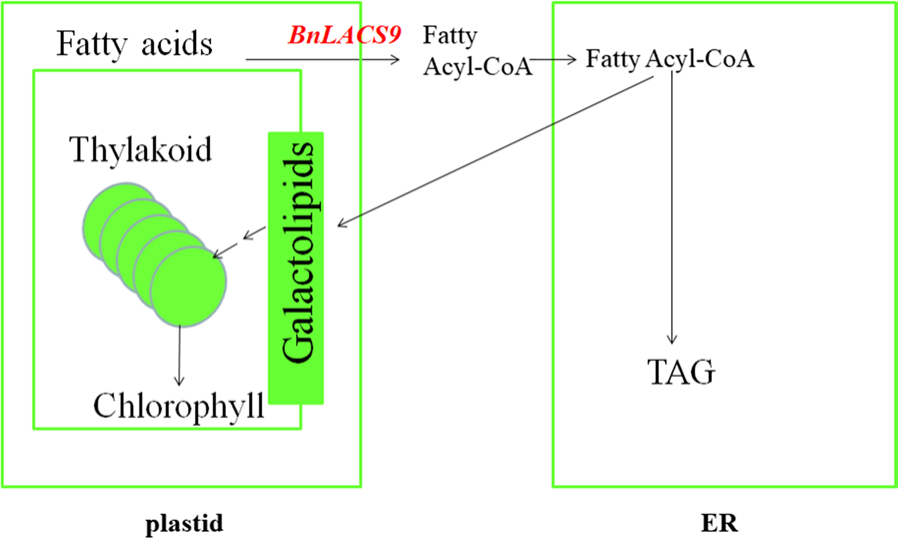
The model of the function of *BnLACS9*. BnLACS9 was located in the chloroplast envelope, which could promote the transport of fatty acid acetyl-CoA from the chloroplast to the ER. In turn, the increased fatty acyl-CoA in ER can promote the content of galactolipids in chloroplasts, increase the number of thylakoid layer structures and improve the photosynthetic efficiency in the chloroplast, and finally increase the synthesis of TAG

In general, BnLACS9 was located in the chloroplast envelope, which could promote the transport of fatty acyl-CoA from the chloroplast to the ER. In turn, the increased fatty acyl-CoA in ER can promote the content of galactolipids in chloroplasts, increase the number of thylakoid grana slice layers and improve the photosynthetic efficiency in the chloroplast, and finally increase the synthesis of TAG (Fig. 11). Therefore, BnLACS9 could increase the oil content of rape seeds.

## Experimental procedures

### Plant materials and growth conditions

The seeds of WT rapeseed cultivar ‘Ningyou 12 (NY12)’ and ‘Zhongshuang 11 (ZS11)’ as well as *N. benthamiana* stored in our lab were sown in a soil mix (peat moss/perlite/vermiculite, 5/3/2, v/v/v) in flower pots (4–5 seedlings/pot) and grown in a plant growth room under the following growth conditions: 22 ± 2 °C with a 16 h light: 8 h dark photoperiod at a light intensity of 5000 LX and 60% relative humidity.

### Sequence analysis

The amino acid sequences of *B. napus* were retrieved through a database search using the amino acid sequences of *Arabidopsis thaliana* LACS proteins: AtLACS1 (AT2G47240), AtLACS2 (AT1G49430), AtLACS3 (AT1G64400), AtLACS4 (AT4G23850), AtLACS5 (AT4G11030), AtLACS6 (AT3G05970), AtLACS7 (AT5G27600), AtLACS8 (AT2G04350) and AtLACS9 (AT1G77590). These sequences were aligned by Clustal-W, and a neighbor-joining tree was built using MEGA version 4.0 [48]. The LACS protein sequences were analyzed using the MEME software [49]. Domains analyzes were performed in Pfam domains database (http://pfam.sanger.ac.uk/search/sequence).

### RNA extraction, cDNA synthesis, and real-time PCR (RT-PCR)

Total RNA extractions from various tissues and reverse transcription were performed according to Wang et al*.* with some modifications [50]. For RT-PCR, 25 ng of cDNA was applied using corresponding gene-specific primer pairs. *BnACTIN* was amplified as the control. RT-PCR was performed on a cycler apparatus (Bio-Rad, USA) using the SYBR Green Master Mix (Vazyme, China) according to the manufacturer’s instructions. Amplification was conducted in 96-well optical reaction plates with the following protocol: 94 ℃ for 4 min, 40 cycles of 94 ℃ for 15 s, 58 ℃ for 15 s, and 72 ℃ for 15 s. The relative expression levels were estimated using the 2^−∆∆CT^ method of Livak and Schmittgen [51]. The RT-PCR was repeated with three biological replications. Primers used in the study are listed in Additional file 8: Table S4.

For transcriptome analysis, 100 mg of the leaf was used for RNA extraction. The purified RNA was detected and qualified using a OneDrop OD-1000 + spectrophotometer (RockGene, Shanghai, China). Library construction was performed according to the standard protocol. The sequencing of the library was performed using the BGISeq-500 platform. The experiment was repeated three times with independent samples. The raw data were filtered to trim adaptor sequences and to remove low-quality sequences (Q < 20) with  > 10% uncertain (N) bases. The NCBI database was used to annotate gene function. Differentially expressed genes (DEGs) were screened according to the NOIseq method [52]. Transcripts that reach the criterion of log_2_-fold change  ≥ 1 (or ≤ -1) and probability  ≥ 0.8 were selected as DEGs.

### Subcellular localization of BnLACS9 by transient expression in N. benthamiana leaves and B. napus cotyledons

To determine the expression location of the BnLACS9 protein, the full-length *BnLACS9* cDNA was PCR amplified using the primers (*BnLACS9*-LF/*BnLACS9*-LR) with the cDNA as a template. The PCR product was cloned into the pENTR™ TOPO® vector (Invitrogen, USA), and the clone with correct sequencing was recombined into pK7WG2.0 by Gateway cloning (Invitrogen, USA) to produce GFP fusion proteins. The recombinant plasmid was transformed into the *Agrobacterium tumefaciens* strain GV3101. Overnight cultured *A. tumefaciens* strain GV3101 harboring the recombinant vectors were washed with sterile 10 mM MMA (10 mM MES, 10 mM MgCl2, and 100 mM acetosyringone, pH 5.6) and suspended with injection buffer MMA to the OD600 = 1.6. The GV3101 containing the construct 35S::p19 was also cultured and suspended with MMA to the OD600 = 1.6, which expression the Tomato bushy stunt virus p19 protein to suppress the gene silencing [53]. The two cultures were mixed 1:1 to the OD600 = 0.8, and followed by incubation at 28 °C with shaking for 3 h. *Agrobacterium* infiltration into 3- to 4-week-old *N. benthamiana* leaves was performed as described previously [54, 55]. Plant culture conditions are the same as the *N. benthamiana* culture conditions as follow: illumination weekly 12 h (light intensity 5000 LX, temperature 24 °C, humidity 60%) and dark 12 h (temperature 22 °C, humidity 60%). An important step is that the injected *B. napus* cotyledons must be maintained humidity in a film bag. The protoplasts from *N. benthamiana* leaves were performed as described previously [41]*.* Four to seven leaf disks with a diameter of 0.8 cm of transfected were cut with a cork borer and transferred into a 10-ml syringe containing 2 ml of cell wall digestion solution (CWDS: 1.5% cellulase R-10, 0.4% macerozyme R-10, 0.4 M mannitol, 20 mM KCl, 20 mM MES (pH 5.6), 10 mM CaCl2, 0.1% BSA). Then, protoplasts and plant tissue were monitored by confocal microscopy (Leica TCS SP5, Wetzlar, Germany). The fluorescence emissions were at 510–540 nm for eGFP and 658–665 nm for chloroplast.

### Expression of BnLACS9 cDNAs in yeast

To generate the recombinant plasmids of pYES2-*BnLACS9*, the primers (*BnLACS9*-YF/*BnLACS9*-YR) were used to amplify the *BnLACS9*, then insert the sequenced *BnLACS9* gene into the multiple cloning sites (*Bam*H I/*Kpn* I) of pYES2 to obtained the recombinant plasmids of pYES2-*BnLACS9*. The recombinant plasmids were transformed into the competent *E. coli*. Then the recombinant plasmid DNA from the identified bacterial colonies was transformed into yeast *YB525* cells (http://www.atcc.org/). The *YB525* containing the recombinant plasmids of pYES2-*BnLACS9* was identified and representative colonies were chosen and grown until mid- to late-log phase in drop-out base and using long-chain FAs 12:0 lauric acid, 14:0 myristic acid, 16:0 palmitic acid, 18:0 stearic acid, 18:1 oleic acid and 22:1 erucic acid as a sole carbon source liquid medium. After 84 h at 30 °C, the growth ratio was determined by a spectrophotometer at OD600.

### Plant expression vector constructs and A. tumefaciens-mediated plant transformations

To construct the expression vector containing the *BnLACS9*, the full-length *BnLACS9* cDNA was PCR amplified using the primers (*BnLACS9*-LF and *BnLACS9*-NR) with the cDNA as a template. The PCR product was cloned into the pENTR™ TOPO® vector (Invitrogen, USA), and the clone with correct sequencing was recombined into the vector pB2WG7.0 by the gateway (Invitrogen, USA) to create the vector under the control of the constitutive *Cauliflower mosaic* virus (CaMV) 35S promoter. The expression vector contained in the *BnLACS9* was transformed into the *A. tumefaciens* strain GV3101. For transient expression in *N. benthamiana* leaves, *Agrobacterium* infiltration into 3- to 4-week-old *N. benthamiana* leaves was performed as described previously [54, 55].

To construct a vector for the constitutive expression of *BnLACS9*, a full-length *BnLACS9* (*BnLACS9*-OF/*BnLACS9*-OR) cDNA was PCR amplified from its cDNA clone, and the CaMV 35S promoter (CaMV 35S-F/CaMV 35S-R) and CaMV Nos terminator (CaMV Nos-F/CaMV Nos-R) was generated from the vector pEGAD. Then, all three products were inserted into the *Eco*R I/*Hin*d III sites of pCAMBIA1300, creating the pCAMBIA1300-35S-*BnLACS9*-NOS vector, which was then transformed into GV3101. To construct the vector to suppress *BnLACS9* expression, a highly conserved 164-bp cDNA fragment of the 5′-open reading frame was amplified with primers *Bnlacs9*-F and *Bnlacs9*-R. This cDNA fragment was subcloned into the pENTR/D-TOPO vector, creating pENTR::*Bnlacs9*. Then, the pENTR::*Bnlacs9* plasmid was transferred into the destination vector pHellsgate 12 to generate pHellsgate 12::*Bnlacs9* using the Gateway LR recombinase (Invitrogen, USA). The plasmid pHellsgate 12::*Bnlacs9* was transformed into GV3101. And the two vectors were transformed using an *Agrobacterium*-mediated transformation method as described by Wang et al*.* [56]. At least three independent transformants were examined as described in the results section. Hygromycin‐resistant T2 generation plants were identified by PCR from the progeny of the primary transformants.

### Ultrastructural observation of chloroplasts

The leaves of wild type and *BnLACS9-*overexpressed lines were cut into 2-mm segments and fixed in 2.5% glutaraldehyde solution. Then they were put into the refrigerator at 4 °C for 24 h. The sample was replaced every 3 days during storage. Fixed samples were sent to the Electron Microscope Center of Nanjing Academy of Agricultural Sciences for sample preparation, and the ultrastructure of chloroplasts was observed by transmission electron microscopy (TEM). TEM sample preparation methods: samples were fixed in 3% glutaraldehyde and 0.1 mol/L phosphate buffer (pH 7.2) for 4 h after collection, then fixed in 1% osmium acid (pH 7.2) for 4 h after washing, and then embedded and polymerized with SPURR after dehydration of acetone step by step. After the dehydration of ethanol step by step, the embedded samples were sliced and then dried at the critical point with by isoamyl acetate. The samples were observed and photographed by JEM-1230 transmission electron microscopy (JEOL LTD, Japan).

### Fatty acyl-CoAs, fatty acid, TAG, and galactolipids analyses

The fatty acyl-CoAs fatty acid, TAG and galactolipids were measured using the method of Larson and Graham (2001) and Larson et al. (2002) [57, 58].

### Chlorophyll determination

Leaves were crushed into powder in liquid nitrogen and then homogenized in 80% acetone, and the debris was removed by centrifugation at 12,000 rpm for 5 min. The absorbance of the supernatant at 663 and 645 nm was measured using a spectrophotometer (GE Healthcare, USA). The chlorophyll A and chlorophyll B concentration of the samples was determined as described previously [59].

### Photosynthetic activity

Photosynthetic parameters of the plants were measured using a portable photosynthesis system (LI-6400XT, LI-COR, http://www.licor.com/) in the field. Measurements were performed in the morning (9:00, 11:00 AM and 15:00 PM). All data represent the means obtained from five plants in one line.

### Seed oil content

Seed oil content was determined using a Foss NIR Systems 5000 near-infrared reflectance spectroscope (Foss NIR Systems Inc., http://www.fossnirsystems.com) and nuclear magnetic resonance (CNMR-1000, China).

### Thin-layer chromatography (TLC) analysis

Total lipid extracts were analyzed by thin-layer chromatography (TLC) on silica gel 60A plates (Merck, 20 × 10 cm, layer thickness 0.2 mm). The plates were washed twice with chloroform/methanol (1:1, by volume) and activated at 180 °C before use. Polar lipids were eluted with Solvent A (chloroform/methanol/acetic acid/water 85:15:10:3.5, by volume); the neutral lipids were separated by TLC in Solvent B (hexane/diethyl ether/acetic acid, 70:30:1, by volume).

Lipid detection was carried out by spraying the plate with 5% sulfuric acid in the water, followed by charring at 180 °C for 5 min, or exposing the TLC plate to iodine vapor, for staining all classes of lipids. The estimation of the content of individual polar and neutral lipids of the total lipid extracts was performed by video densitometry analysis of spots on TLC, obtained after averaging three replicates of C, NI, and NII (ImageJ software).

## Supplementary Information

**Additional file 1: Figure S1.** The growth conditions of the yeast of *YB525* contained the *BnLACS9*-pYES2 and the empty vector of pYES2. The yeasts were cultured in the liquid medium which fatty acid C22:1, C18:0, C16:0, C18:1, C14:0, and C12:0 were used as the sole carbon source. * *p* < 0.05, ** *p* < 0.01, *** *p* < 0.001. Student’s *t*-test was used to generate the *p*-value.

**Additional file 2: Figure S2.** Expression profiles of the *BnLACS9* in the *Brassica napus* of Ningyou 12. The tissues that have been analyzed include root, stem, young leaf, old leaf, flower, and silique.

**Additional file 3: Figure S3.** The identification of *BnLACS9* overexpression transgenic plants. (A) The *BnLACS9 *overexpression vector and identification of *Brassica napus* plants transgenic pCAMBIA1300-35S-*BnLACS9*-NOS by PCR. F and R are the primers used to identify the transgenic plants. Line 1: 5000 DNA Maker; Line 2 to Line 21: *BnLACS9* overexpression transgenic lines; Line 22: PCR result using the plasmid pCAMBIA1300-35S-*BnLACS9-*NOS as positive control; line23-24: negative control. (B) RT-PCR analysis of *BnLACS9* overexpression transgenic plants. NY12: Wild type; The *BnLACS9* overexpression lines (Over-*BnLACS9*-6, Over-*BnLACS9*-12, Over-*BnLACS9*-13, Over-*BnLACS9*-17, Over-*BnLACS9*-18). *BnACTIN* was used as an internal control. The data show means ± standard errors (N = 3). * *p* < 0.05, ** *p *< 0.01, *** *p* < 0.001. Student’s t-test was used to generate the *p*-value.

**Additional file 4: Figure S4.** The content of the lipids. Line 1-line 5: *BnLACS9-6*, *BnLACS9-12*, *BnLACS9-13*, *BnLACS9-17*, *BnLACS9-18*; Line 6: NY12 (CK).

**Additional file 5: Table S1.** A complete list of 29 identified BnaLACS in the study

**Additional file 6: Table S2.** The CoA content in *N. benthamiana* leaves.

**Additional file 7: Table S3.** Rapeseed oil content was determined by NIR (Near-infrared)

**Additional file 8: Table S4.** The primers used in the experiment.

## Data Availability

The datasets used and/or analyzed during the current study are available from the corresponding author upon reasonable request.
