# Supplementary figures and images for: Overexpression the BnLACS9 could increase the chlorophyll and oil content in Brassica napus
Source: Biotechnol Biofuels Bioprod. 2023 Jan 6;16:3. doi: 10.1186/s13068-022-02254-3 (PMC9825004; doi:10.1186/s13068-022-02254-3)

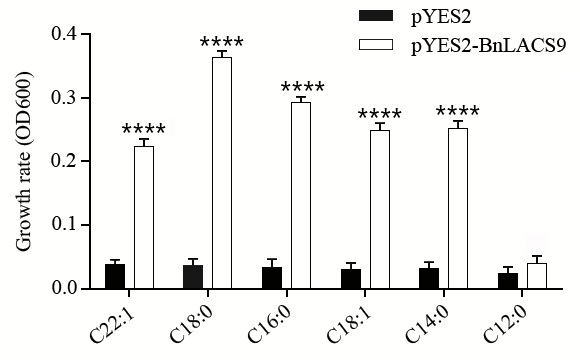

Supplement: Supplementary file 1 — Additional file 1: Figure S1. The growth conditions of the yeast of YB525 contained the BnLACS9-pYES2 and the empty vector of pYES2. The yeasts were cultured in the liquid medium which fatty acid C22:1, C18:0, C16:0, C18:1, C14:0, and C12:0 were used as the sole carbon source. * p < 0.05, ** p < 0.01, *** p < 0.001. Student’s t-test was used to generate the p-value. [file 13068_2022_2254_MOESM1_ESM.tif]

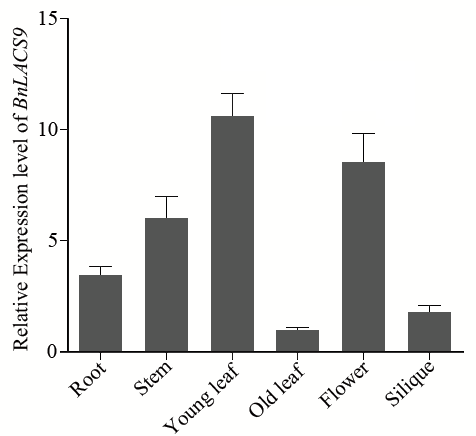

Supplement: Supplementary file 2 — Additional file 2: Figure S2. Expression profiles of the BnLACS9 in the Brassica napus of Ningyou 12. The tissues that have been analyzed include root, stem, young leaf, old leaf, flower, and silique. [file 13068_2022_2254_MOESM2_ESM.tif]

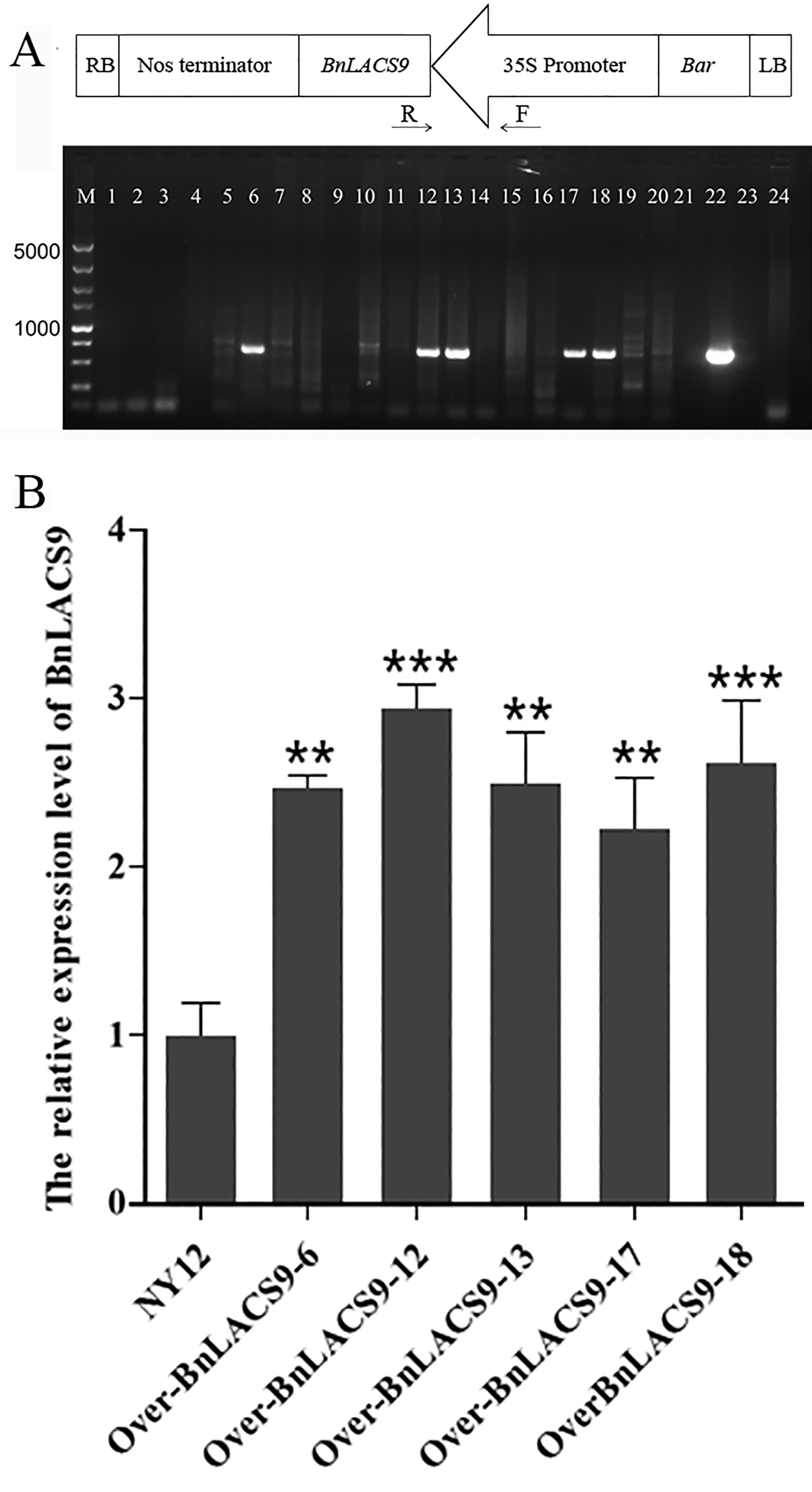

Supplement: Supplementary file 3 — Additional file 3: Figure S3. The identification of BnLACS9 overexpression transgenic plants. (A) The BnLACS9 overexpression vector and identification of Brassica napus plants transgenic pCAMBIA1300-35S-BnLACS9-NOS by PCR. F and R are the primers used to identify the transgenic plants. Line 1: 5000 DNA Maker; Line 2 to Line 21: BnLACS9 overexpression transgenic lines; Line 22: PCR result using the plasmid pCAMBIA1300-35S-BnLACS9-NOS as positive control; line23-24: negative control. (B) RT-PCR analysis of BnLACS9 overexpression transgenic plants. NY12: Wild type; The BnLACS9 overexpression lines (Over-BnLACS9-6, Over-BnLACS9-12, Over-BnLACS9-13, Over-BnLACS9-17, Over-BnLACS9-18). BnACTIN was used as an internal control. The data show means ± standard errors (N = 3). * p < 0.05, ** p < 0.01, *** p < 0.001. Student’s t-test was used to generate the p-value. [file 13068_2022_2254_MOESM3_ESM.tif]

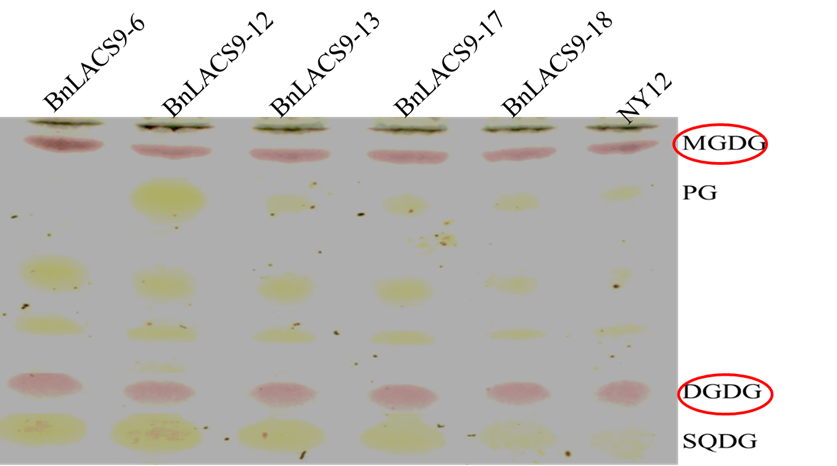

Supplement: Supplementary file 4 — Additional file 4: Figure S4. The content of the lipids. Line 1-line 5: BnLACS9-6, BnLACS9-12, BnLACS9-13, BnLACS9-17, BnLACS9-18; Line 6: NY12 (CK). [file 13068_2022_2254_MOESM4_ESM.tif]
