# Supplementary material for: Overexpression the BnLACS9 could increase the chlorophyll and oil content in Brassica napus
Source: Biotechnol Biofuels Bioprod. 2023 Jan 6;16:3. doi: 10.1186/s13068-022-02254-3 (PMC9825004; doi:10.1186/s13068-022-02254-3)
Supplement: Supplementary file 5 — Additional file 5: Table S1. A complete list of 29 identified BnaLACS in the study [file 13068_2022_2254_MOESM5_ESM.doc]

**Table 1** A complete list of 29 identified BnaLACSs in the study

| Isoforms | Gene name | At Orthologs | Location | gDNA size (bp) | Exon & Intron | CDS size (nts) | Peptide residues | Theoreti- cal pI | Theoretical Mw (Da) |
| --- | --- | --- | --- | --- | --- | --- | --- | --- | --- |
| LACS1 | BnaA05g00630D | AT2G47240.1 | chrA05:304,482..307,202 | 2721 | 10&9 | 1296 | 431 | 6 | 49,011.27 |
|  | BnaA05g00640D | AT2G47240.1 | chrA05:369,047..373,989 | 4943 | 7&6 | 882 | 293 | 5.38 | 33,121.48 |
|  | BnaC03g25940D | AT2G47240.1 | chrC03:14,648,077..14,654,186 | 6110 | 20&19 | 2058 | 685 | 8.56 | 77,815.5 |
|  | BnaC04g00320D | AT2G47240.1 | chrC04:130,463..133,183 | 2721 | 10&9 | 1296 | 431 | 6 | 49,011.27 |
|  | BnaC04g51420D | AT2G47240.1 | chrC04:48,710,206..48,715,999 | 5794 | 17&16 | 1983 | 660 | 6.33 | 74,496.94 |
| LACS2 | BnaA05g16170D | AT1G49430.1 | chrA05:10,774,154..10,779,061 | 4908 | 19&18 | 2001 | 666 | 5.98 | 74,373.26 |
|  | BnaC05g51350D | AT1G49430.1 | chrC05:2,357,024..2,363,526 | 6503 | 19&18 | 2001 | 666 | 6.21 | 74,269.16 |
| LACS3 | BnaC09g11030D | AT1G64400.1 | chrC09:7,630,515..7,635,842 | 5328 | 21&20 | 2361 | 786 | 6.25 | 88,342.56 |
| LACS4 | BnaA01g13470D | AT4G23850.1 | chrA01:6,850,542..6,855,021 | 4480 | 18&17 | 2004 | 667 | 5.96 | 74,462.85 |
|  | BnaC01g15670D | AT4G23850.1 | chrC01:10,752,424..10,756,922 | 4499 | 18&17 | 2004 | 667 | 5.96 | 74,464.89 |
| LACS5 | BnaA02g21860D | AT4G11030.1 | chrA02:14,250,317..14,254,300 | 3984 | 20&19 | 1980 | 659 | 6.05 | 73,395.55 |
|  | BnaA09g22150D | AT4G11030.1 | chrA09:14,727,049..14,730,917 | 3869 | 18&17 | 2001 | 666 | 6.25 | 74,288.4 |
|  | BnaC02g28920D | AT4G11030.1 | chrC02:28,320,133..28,324,178 | 4046 | 20&19 | 1986 | 661 | 5.96 | 73,452.51 |
|  | BnaC09g26090D | AT4G11030.1 | chrC09:25,876,013..25,880,576 | 4564 | 19&18 | 2202 | 733 | 5.98 | 81,506.48 |
| LACS6 | BnaA03g29320D | AT3G05970.1 | chrA03:14,247,032..14,252,281 | 5250 | 23&22 | 2091 | 696 | 7.56 | 76,202.95 |
|  | BnaA05g31340D | AT3G05970.1 | chrA05:21,610,343..21,615,685 | 5343 | 23&22 | 2064 | 687 | 7.57 | 75,321.96 |
|  | BnaC03g34500D | AT3G05970.1 | chrC03:20,903,411..20,908,669 | 5259 | 23&22 | 2091 | 696 | 7.56 | 76,243.01 |
|  | BnaC05g45860D | AT3G05970.1 | chrC05:41,607,925..41,613,374 | 5450 | 23&22 | 2064 | 687 | 7.57 | 75,318.94 |
| LACS7 | BnaA06g28680D | AT5G27600.1 | chrA06:19,660,381..19,664,851 | 4471 | 23&22 | 2100 | 699 | 6.72 | 77,023.14 |
|  | BnaC07g28060D | AT5G27600.1 | chrC07:33,371,838..33,376,153 | 4316 | 23&22 | 2100 | 699 | 6.31 | 77,207.29 |
| LACS8 | BnaA01g23830D | AT2G04350.1 | chrA01:16,236,739..16,237,896 | 1158 | 2&1 | 207 | 69 | 7.97 | 7,735.86 |
|  | BnaA03g57930D | AT2G04350.1 | chrA03:1,374,321..1,378,991 | 4671 | 11&10 | 2166 | 721 | 7.12 | 78,587.38 |
|  | BnaA05g07920D | AT2G04350.1 | chrA05:4,352,555..4,354,626 | 2072 | 4&3 | 732 | 243 | 9.65 | 27,492.68 |
|  | BnaA10g24190D | AT2G04350.1 | chrA10:15,825,003..15,828,125 | 3123 | 8&7 | 846 | 281 | 9.53 | 31,463.72 |
|  | BnaC03g44430D | AT2G04350.1 | chrC03:29,547,508..29,550,894 | 3387 | 11&10 | 2151 | 716 | 8.11 | 78,022.72 |
|  | BnaC04g26780D | AT2G04350.1 | chrC04:28,080,319..28,085,567 | 5249 | 7&6 | 1053 | 350 | 8.23 | 38,531.81 |
| LACS9 | BnaC06g20910D | AT1G77590.1 | chrC06:22,958,082..22,961,638 | 3557 | 10&9 | 2082 | 693 | 6.25 | 75,991.46 |
|  | BnaA07g20920D | AT1G77590.1 | chrA07:16,300,672..16,304,048 | 3377 | 10&9 | 2082 | 693 | 6.25 | 75,991.46 |
