# Supplementary material for: Overexpression the BnLACS9 could increase the chlorophyll and oil content in Brassica napus
Source: Biotechnol Biofuels Bioprod. 2023 Jan 6;16:3. doi: 10.1186/s13068-022-02254-3 (PMC9825004; doi:10.1186/s13068-022-02254-3)
Supplement: Supplementary file 6 — Additional file 6: Table S2. The CoA content in N. benthamiana leaves. [file 13068_2022_2254_MOESM6_ESM.docx]

Table S2 The CoA content in *N. benthamiana* leaves

| Acyl-CoA | P19  CoA content  (avfmol/mg) | ±SD | pB2GW7.0-*BnLACS9*  CoA content  (avfmol/mg) | | ±SD |
| --- | --- | --- | --- | --- | --- |
| C2:0 | 6.61083 | 1.39379 | | 7.1831 | 1.358259 |
| C4:0 | 7.387154 | 6.789183 | | 14.32233 | 0.97188 |
| C6:0 | 8.322458 | 2.74276 | | 6.547055 | 1.406957 |
| C8:0 | 36.06325 | 6.266329 | | 42.23341 | 5.334781 |
| C10:0 | 0.647754 | 0.567659 | | 0.869375 | 0.580957 |
| C12:0 | 1.564231 | 0.299744 | | 0.770367 | 0.516654 |
| C14:0 | 10.88591 | 0.777906 | | 12.78282 | 0.763995 |
| C18:3 | 47.59071 | 6.161552 | | 41.7954 | 6.390999 |
| C16:1 | 4.173502 | 1.654766 | | 7.576914 | 0.711642 |
| C18:2 | 23.56197 | 3.008565 | | 59.2165 | 9.703042 |
| C16:0 | 134.9407 | 7.264136 | | 221.6034 | 14.0447 |
| C18:1 | 6.365882 | 0.315986 | | 7.941865 | 1.109978 |
| C20:2 | 0.967406 | 0.186929 | | 1.215209 | 0.579532 |
| C18:0 | 30.6479 | 0.65538 | | 36.49893 | 1.843828 |
| C20:1 | 24.87717 | 1.749918 | | 45.81828 | 3.616426 |
| C20:0 | 12.76891 | 0.180914 | | 16.41325 | 0.587022 |
| C22:1 | 26.27505 | 0.531116 | | 38.57146 | 1.448839 |
| C22:0 | 11.81541 | 0.246871 | | 13.21715 | 0.53581 |
| TOTAL | 395.4662 | 13.63986 | | 574.5768 | 35.79813 |
