# Supplementary material for: Overexpression the BnLACS9 could increase the chlorophyll and oil content in Brassica napus
Source: Biotechnol Biofuels Bioprod. 2023 Jan 6;16:3. doi: 10.1186/s13068-022-02254-3 (PMC9825004; doi:10.1186/s13068-022-02254-3)
Supplement: Supplementary file 7 — Additional file 7: Table S3. Rapeseed oil content was determined by NIR (Near-infrared) [file 13068_2022_2254_MOESM7_ESM.docx]

Table S3 Rapeseed oil content (%) was determined by NIR (Near-infrared)

| Lines | Oleic acid | Linoleic acid | Linolenic acid | Erucic acid | Saturated fatty acids | Glucosinolate | Oil | Protein | Water |
| --- | --- | --- | --- | --- | --- | --- | --- | --- | --- |
| *NY12* | 59.14 | 23.43 | 9.18 | 1.13 | 6.61 | 41.45 | 39.70 | 25.24 | 8.40 |
| *BnLACS9-6* | 54.87 | 21.71 | 8.31 | 1.94 | 6.76 | 17.88 | 43.34 | 22.67 | 9.53 |
| *BnLACS9-12* | 53.84 | 21.90 | 10.04 | 1.07 | 6.57 | 11.31 | 45.64 | 22.00 | 8.92 |
| *BnLACS9-18* | 55.72 | 22.46 | 8.71 | 0.22 | 6.45 | 11.52 | 43.86 | 23.71 | 8.99 |
