# Supplementary material for: Overexpression the BnLACS9 could increase the chlorophyll and oil content in Brassica napus
Source: Biotechnol Biofuels Bioprod. 2023 Jan 6;16:3. doi: 10.1186/s13068-022-02254-3 (PMC9825004; doi:10.1186/s13068-022-02254-3)
Supplement: Supplementary file 8 — Additional file 8: Table S4. The primers used in the experiment. [file 13068_2022_2254_MOESM8_ESM.docx]

Table S4 The primers used in the experiment

| Primer | Sequence |
| --- | --- |
| *BnLACS9*-RTF | 5'-ATTGGGCACAAGTCTGAGG-3' |
| *BnLACS9*-RTR | 5'-TGACCTCTGTCTCATTAAGCG-3' |
| *BnACTIN*-RTF | 5'-ATGGCCGATGGTGAGGACATTC-3' |
| *BnACTIN*-RTR | 5'-GGTGCGACCACCTTGATCTTC-3' |
| RNAi-BnLACS9-F | 5’-CACCATTGGGCACAAGTCTGAGG-3’ |
| RNAi-BnLACS9-R | 5’-TGACCTCTGTCTCATTAAGCG -3’ |
| *BnLACS9*-YF | 5’-AAggatccATGATTCCTTACGCTGCTGG-3’ (*Bam*H I) |
| *BnLACS9*-YR | 5’-AAggtaccTTAGGAAGCATATAGCTTGGTG-3’ (*Kpn* I) |
| pB2GW7.0-35S-F | 5'-CTTCGCAAGACCCTTCCTC-3' |
| *BnLACS9*-LF | 5'-CACCATGATTCCTTACGCTGCTGG-3’ |
| *BnLACS9*-LR | 5'-GGAAGCATATAGCTTGGTG-3’ |
| *BnLACS9*-NR | 5’-TTAGGAAGCATATAGCTTGGTG-3’ |
| *BnLACS9*-OF | 5’-AAggtaccATGATTCCTTACGCTGCTGG-3’ (*Kpn* I) |
| *BnLACS9*-OR | 5’-AAggatccTTAGGAAGCATATAGCTTGGTG-3’ (*Bam*H I) |
| CaMV 35S-F | 5’-AAgaattcTTAATTAAGAGCTCGCATGCC-3’ (EcoR I) |
| CaMV 35S-R | 5’-AAggtaccGTCCCCGTGTTCTCTCCAA-3’ (*Kpn* I) |
| CaMV Nos-F | 5’-AAggatccGAATTTCCCCGATCGTTCAA-3’ (*Bam*H I) |
| CaMV Nos-R | 5’-AAaagcttGATCTAGTAACATAGATGACACCGC-3’ (*Hin*d III) |
| M13-F | 5´-GTAAAACGACGGCCAG-3 |
| M13-R | 5´-CAGGAAACAGCTATGAC-3 |
| RKAN-F | 5'-CTGATGCCGCCGTGTTC-3' |
| RKAN-R | 5'-CCAGAGTCCCGCTCAGAAGA-3' |
| PH12-F | 5'-ATTAGTCGAACATGAATAAACAAG-3' |
| PH12-R | 5'-TTAAATCATCAAACCAGCTAGAAT-3' |
| qRT*BnATS1*-F | 5'-AATGGTTTTCTGGTGGAATGTA-3' |
| qRT*BnATS1*-R | 5'-ATTCTCCGCCCTCCTGTC-3' |
| qRT*BnCAO*-F | 5'-ATTACCAGTTAGAGTTGCGACAG-3' |
| qRT*BnCAO*-R | 5'-GCAGTGAAAGCAACGGGA-3' |
| qRT*BnCDP*-DAGS-F | 5'-GGGCTGGCTCCAATGTGAT-3' |
| qRT*BnCDP*-DAGS-R | 5'-TGAAACCACTGGCAAAGAAGC-3' |
| qRT*BnCHLD*-F | 5'-CAGAGGCTACTGGGAGACATC-3' |
| qRT*BnCHLD*-R | 5'-GACTCAGCAAGGCAGAAGAAT-3 |
| qRT*BnDGDGS*-F | 5'-GTGGAGGGAGGTGAGGGACT-3' |
| qRT*BnDGDGS*-R | 5'-GACGGAGACGACGACACGAC-3' |
| qRT*BnHEMA*-F | 5'-ATGGACCTTCTCATCTTCTTGTT-3' |
| qRT*BnHEMA*-R | 5'-CCCAAACAATCATTACCCAAG-3' |
| qRT*BnLPAAT*-F | 5'-AGGCTAAATGCGAAAGGAG-3' |
| qRT*BnLPAAT*-R | 5'-TCTAAGAAAGACGGACAACG-3' |
| qRT*BnMGDGS*-F | 5'-TGGGTAATGATTGTTTGGGC-3' |
| qRT*BnMGDGS*-R | 5'-AAGCACCGCATCTGAATCCT-3' |
| qRT*BnPDAT*-F | 5'-CAGATTGATGACGGTCTTGTG-3' |
| qRT*BnPDAT*-R | 5'-GAACTTACGGATTCAACGATTT-3' |
| qRT*BnPGPP*-F | 5'-ATCATTCAATCCGAAACCAA-3' |
| qRT*BnPGPP*-R | 5'-CTCCTGTTCGGTAGTCAAAGTAG-3' |
| qRT*BnPGPS*-F | 5'-AGAACGGTTGTAATGGTGGC-3' |
| qRT*BnPGPS*-R | 5'-CCACCAGCAATCAACGTAGAA-3' |
| qRT*BnPOR*-F | 5'-GCTTCGTCAGGGTTAGGTCTA-3' |
| qRT*BnPOR*-R | 5'-AACTGTCTCACGCTGTCCAAC-3' |
| qRT*BnSLS*-F | 5'-GACTTGAGGTGGTTTCTGCTT-3' |
| qRT*BnSLS*-R | 5'-TAACCTCGTCTCCCATTTCA-3' |
